# Profiling Sirolimus-Induced Inflammatory Syndrome: A Prospective Tricentric Observational Study

**DOI:** 10.1371/journal.pone.0053078

**Published:** 2013-01-07

**Authors:** Fanny Buron, Paolo Malvezzi, Emmanuel Villar, Cécile Chauvet, Bénédicte Janbon, Laure Denis, Maria Brunet, Sameh Daoud, Rémi Cahen, Claire Pouteil-Noble, Marie-Claude Gagnieu, Jacques Bienvenu, François Bayle, Emmanuel Morelon, Olivier Thaunat

**Affiliations:** 1 Service de Transplantation, néphrologie et Immunologie Clinique de l’Hôpital Edouard Herriot, Hospices Civils de Lyon, Lyon, France; 2 Université de Lyon, Lyon, France; 3 Clinique de Néphrologie, Dialyse et Transplantation, CHU Grenoble, Grenoble, France; 4 Département de Néphrologie du Centre Hospitalier Lyon Sud, Hospices Civils de Lyon, Pierre-Bénite, France; 5 Laboratoire d’Immunologie du Centre Hospitalier Lyon Sud, Hospices Civils de Lyon, Pierre-Bénite, France; 6 Laboratoire de Pharmacologie de l’Hôpital Edouard Herriot, Hospices Civils de Lyon, Lyon, France; 7 Unit 851, Institut National de la Santé et de la Recherche Médicale, Lyon, France; University of Leuven, Rega Institute, Belgium

## Abstract

**Background:**

The use of the immunosuppressant sirolimus in kidney transplantation has been made problematic by the frequent occurrence of various side effects, including paradoxical inflammatory manifestations, the pathophysiology of which has remained elusive.

**Methods:**

30 kidney transplant recipients that required a switch from calcineurin inhibitor to sirolimus-based immunosuppression, were prospectively followed for 3 months. Inflammatory symptoms were quantified by the patients using visual analogue scales and serum samples were collected before, 15, 30, and 90 days after the switch.

**Results:**

66% of patients reported at least 1 inflammatory symptom, cutaneo-mucosal manifestations being the most frequent. Inflammatory symptoms were characterized by their lability and stochastic nature, each patient exhibiting a unique clinical presentation. The biochemical profile was more uniform with a drop of hemoglobin and a concomitant rise of inflammatory acute phase proteins, which peaked in the serum 1 month after the switch. Analyzing the impact of sirolimus introduction on cytokine microenvironment, we observed an increase of IL6 and TNFα without compensation of the negative feedback loops dependent on IL10 and soluble TNF receptors. IL6 and TNFα changes correlated with the intensity of biochemical and clinical inflammatory manifestations in a linear regression model.

**Conclusions:**

Sirolimus triggers a destabilization of the inflammatory cytokine balance in transplanted patients that promotes a paradoxical inflammatory response with mild stochastic clinical symptoms in the weeks following drug introduction. This pathophysiologic mechanism unifies the various individual inflammatory side effects recurrently reported with sirolimus suggesting that they should be considered as a single syndromic entity.

## Introduction

Sirolimus is the first identified member of a new family of potent immunosuppressants that act by inhibiting mammalian target of sirolimus (mTOR), thereby inducing cell cycle blockade at the G1 to S transition. By blocking lymphocyte proliferation upon cytokine engagement, sirolimus efficiently prevents transplant rejection [1] allowing early dose reduction of the nephrotoxic calcineurin inhibitors (CNI). Furthermore, sirolimus interferes with fibrotic processes that characterize chronic allograft nephropathy [2], [3] and influences the preferential development of immunological tolerance in experimental models [4]–[8]. Another interesting feature is that the mTOR pathway is central for vital aspects of tumor development, including angiogenesis and cell growth. Sirolimus has therefore anticancer activities [1], [9], which may prove critical to prevent this life threatening complication in transplant recipients.

Despite its promising profile, enthusiasm for the drug faded with large trials showing a very high discontinuation rates (up to 50%) due to frequent adverse effects [10], [11]. Sixty percent of patients receiving mTOR inhibitors require lipid-lowering therapy to control hypercholesterolemia, and these drugs also significantly increase the risk for post-transplant diabetes. Antiproliferative property of sirolimus induces myelosuppression, infertility [12], and impairs wound healing, which in turn translates into a higher incidence of wound dehiscence, lymphoceles, and a longer time for recovery after tubular necrosis [10]. Moreover, while mTOR inhibitors are often classified as nonnephrotoxic, several studies have reported that they can induce proteinuria and predispose to focal segmental glomerulosclerosis lesions through direct toxic effects on podocyte [13], [14]. Finally, a striking characteristic of the safety profile of sirolimus is the frequent occurrence of a wide range of inflammatory manifestations, which have a strong negative impact on the tolerance to the drug. Among the best characterized sirolimus-induced inflammatory symptoms are: stomatitis [15], inflammatory skin disorders (including rash and acnea); [15]), arthritis [16], colitis with abdominal pain and diarrhea [17], and pneumonitis [18].

Besides clinical inflammatory manifestations, sirolimus also induce biochemical evidence of a chronic inflammatory state [19].

The occurrence of inflammatory side effects after the introduction of an immunosuppressive drug is somewhat paradoxical and the pathophysiology of these inflammatory adverse events has remained elusive.

We undertook the prospective tricentric SIRolimus Inflammation LYon GREnoble (SIRILYGRE) study to characterize more precisely the clinical and biological profiles of sirolimus-induced inflammatory syndrome and to gain insight into its pathophysiology.

## Patients and Methods

### Ethics Statement

SIRILYGRE is a multicentric prospective observational study approved by an Institutional Review Board (CPP Sud-Est IV). All the patients enrolled gave their written informed consent and the investigations have been conducted according to the principles expressed in the Declaration of Helsinki.

The extra costs due to cytokine dosages were covered by a grant from Wyeth Laboratory. This commercial funder did not play any part in the design of the study, the interpretation of the results, and the redaction of the manuscript. This funding did not alter in any mean our adherence to all the PLoS ONE policies on sharing data and materials.

### Study Population

Patients enrolled in the SIRILYGRE study were kidney transplant recipients followed in Hôpital Edouard Herriot (Lyon), Centre Hospitalier Lyon Sud (Lyon) or Hôpital Universitaire de Grenoble.

Eligible patients were adult transplanted for more than 3 months with stable graft function, who required a switch from calcineurin inhibitor to sirolimus-based immunosuppression whatever the indication. Characteristics of the population are presented in **Table 1**.

**Table 1:** Characteristics of the population.

Number of patient	30
**Age** (years)	55±12
**Gender** (male)	24 (80%)
**BMI** (kg/m^2^)	25±4
**Ethnicity**	
Caucasian	27 (90%)
Black	2 (7%)
Asiatic	1 (3%)
**Initial nephropathy**	
polycystic kidney disease	10 (33%)
IgA nephropathy	6 (20%)
glomerulopathy	3 (10%)
vascular disease	3 (10%)
malformative uropathy	2 (7%)
other*	4 (13%)
unknown	2 (7%)
**Rank of transplantation**	
first	29 (97%)
second	1 (3%)
**Panel reactive antibodies (%)**	0
**Type of donation**	
deceased donor	29 (97%)
living donor	1 (3%)
**Donor age** (years)	49±15
**HLA mismatches**	3.9±1.3
**Delay post transplantation (years)**	4.0±5.1
**Switch indication**	
post transplant neoplasia	11 (37%)
chronic allograft nephropathy	8 (27%)
systematic switch	6 (20%)
polycystic kidney disease	3 (10%)
CNI intolerance	2 (7%)
**eGFR** (ml/min/1.73 m^2^)	54±17
**Genotype of cytochrome 3A5** ♦	
*1/*1	2 (7%)
*1/*3	6 (20%)
*3/*3	22 (73%)

Data are given as mean ± SD or number (%).

* Other initial nephropathies were: Wegener’s granulomatosis, diabetic nephropathy, CNI toxicity, posttraumatic loss of kidney.

eGFR was estimated at the time of inclusion by MDRD formula.

♦ Genotype of cytochrome 3A5: *1/*1 and *1/*3 are expressors, *3/*3 are non-expressors.

### Design of the Study

SIRILYGRE is a non-randomized before-and-after cohort study.

The clinical and biological parameters of interest were assessed at enrolment, (reference value) and 15, 30 and 90 days after sirolimus introduction.

The repeated measures obtained during the 3 months follow-up period were compared to the individual reference value, which was considered as control for the patient, thereby optimising the statistical power of the analysis.

### Management of the Switch from Calcineurin Inhibitor to Sirolimus-based Immunosuppression

**Figure 1:**
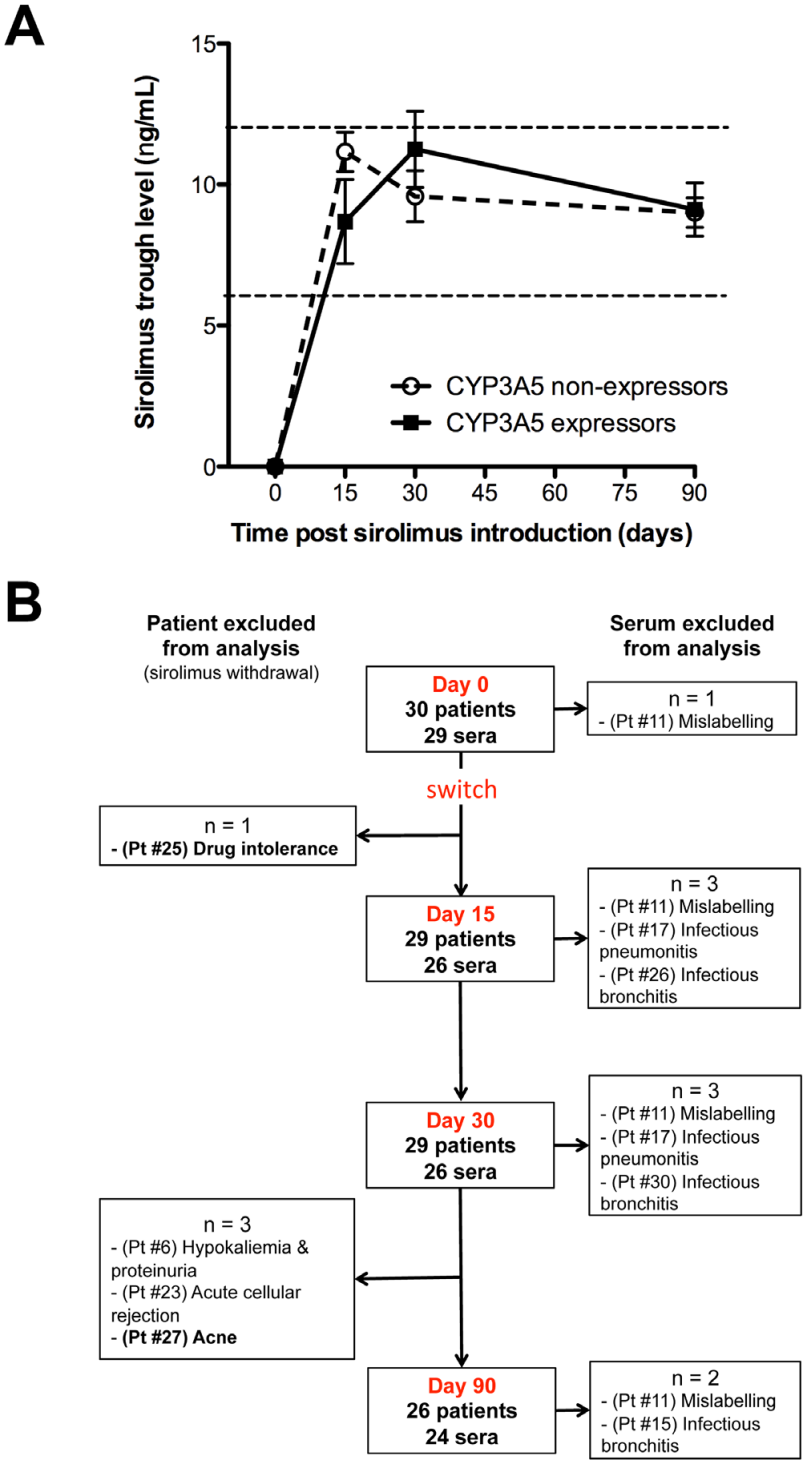
Summary of the data available for analysis. A) Mean ± SEM of sirolimus trough levels are indicated at each time points for CYP3A5 expressors and non-expressors. Dashed lines indicate the target values. B) Flowchart of SIRILYGRE study summarizing data available for analysis. Among the 4 patients (Pt) excluded because of sirolimus discontinuation, 2 (#25 & #27, in bold) experienced severe inflammatory symptoms. Five sera were excluded from analysis because of the presence of a documented confounding inflammatory condition (sepsis). For one patient (#11) no analysis of cytokine dosage could be performed because time point of collection was not indicated on the tubes.

Sirolimus was introduced at a dose of 2 mg daily at noon, and then adapted to trough level (target: 6–12 ng/mL; **Figure 1A**). This relatively large trough level target was set in accordance with previous publication [20] to allow physicians to adapt drug posology to each patients according to his individual immunological risk and time from transplantation.

The percentage of patients with sirolimus trough levels within the target (6–12 ng/mL) at 15, 30, and 90 days were respectively 62%, 72% and 92%, and was not significantly affected by CYP3A5 genotype (**Figure 1A**).

Calcineurin inhibitor posology was reduced by 50% weekly and stopped after one month.

Changes in concomitant immunosuppressive drugs are summarized in **Table 2**. Briefly, mycophenolate mofetil posology was reduced in 2 patients and replaced at the same dose by mycophenolate acid in one because of diarrhea. Prednisolone was followed at the same dose for all but one.

**Table 2:** Changes in concomitant immunosuppressive drugs after sirolimus introduction.

	Mycophenolate Mofetil			Prednisolone		
	Ciclosporine	Tacrolimus	Total	Ciclosporine	Tacrolimus	Total
No treatment	1 (6%)	0	1 (3%)	3 (18%)	5 (38%)	8 (27%)
No change	12 (71%)	9 (69%)	21 (70%)	13 (76%)	6 (46%)	19 (63%)
Decrease in dose	1 (6%)	1 (8%)	2 (7%)	0	0	0
Introduction	0	0	0	0	1 (8%)	1 (3%)
Unknown	3 (18%)	3 (23%)	6 (20%)	1(6%)	1 (8%)	2 (7%)
Total	17	13	30	17	13	30

Results are expressed in number of patients (%).

### Data Management

Clinical and biological data were collected just before the switch (day 0, to determine the baseline level), and 15, 30, and 90 days after sirolimus introduction. Plasma was collected at each time point for cytokines measurement.

Visual analogue scales provide a simple technique for measuring subjective experience. They have been established as valid and reliable in a wide range of clinical applications [21]. The intensity of clinical inflammatory manifestations previously related to sirolimus in the literature [i.e. stomatitis [15], inflammatory skin disorders (rash and acnea; [15]), arthritis [16], and pneumonitis [18]] was therefore measured by the patients using visual analogue scales (0 to 100 mm).

Delta visual analogue scale value was defined as the number obtained when the baseline visual analogue scale value is subtracted from the visual analogue scale value measured at the considered time point.

Delta visual analog scale value of at least 10 mm was considered significant.

Global delta visual analog scale is the sum of the delta visual analog scale value of each inflammatory symptoms of a patient at the considered time point.

Patients for whom sirolimus had to be withdrawn were excluded for the next time points. Patients with a confounding inflammatory condition were excluded from the analysis at the considered time point. The list of the data available for analysis is presented in **Figure 1B** **.**

Of note, cyclosporine withdrawal increases mycophenolic acid exposition, which could promote the occurrence of digestive side effects for patients switched from cyclosporine to sirolimus without adaptation of mycophenolate mofetil dose. Digestive side effects were therefore excluded from the analysis in the present study.

### Genotyping of Cytochrome P450 3A5

Genotyping of cytochrome P450 3A5 (CYP3A5) was performed at inclusion, as described by Anglicheau et al [22]. Briefly, quantitative PCR conducted with primers specific for each allele determined whether the patient was *3/*3 (n = 22, 73%), *3/*1 (n = 6, 20%) or *1/*1 (n = 2, 7%). CYP3A5 expression is significantly lower in individuals with the CYP3A5 *3/*3 genotype (CY3A5 non-expressors) than in CYP3A5 expressors (namely individuals with either the *1/*1 or *1/*3 genotypes).

### Measurement of Plasma Cytokines

EDTA blood tubes were centrifuged at 1500 g for 10 min at 20°C, and plasma was immediately aliquoted and stored at −80°C until analysis.

The determination of plasma cytokines (IL-6, TNFα, IL-10, sTNFR I and II) was centralized and performed for all the samples at the end of the study using commercially available ELISA kits (Invitrogen, Biosource Europe SA, Nivelles, Belgium) according to manufacturer instructions. IL-6 and IL-10 were measured using high sensitivity kits with a detection limit of 0,1 and 0,2 pg/mL respectively. For TNFα, type I and type II soluble receptors, detection limits were 3 pg/mL, 0,05 ng/mL and 0,1 ng/mL respectively. When the measured value was below the detection limit the value was set to zero for calculation and plotting.

### Statistical Analysis

Continuous variables obtained from repeated measures 15, 30 and 90 after the switch were compared to the baseline value using the nonparametric Friedman test with Dunns post test in Prism 5.0 software (GraphPad, La Jolla, CA). Continuous variables were compared using t test when indicated.

Correlations between continuous variables were tested using a linear regression model with JMP 6.0 software (SAS Institute,Cary, NC).

The p-values of <0.05 were considered statistically significant.

## Results

### Stochastic Inflammatory Clinical Manifestations Following Sirolimus Introduction

Thirty renal transplanted patients, who required a change from calcineurin inhibitor to sirolimus-based immunosuppression (**Table 1**), were prospectively followed for 3 months after the switch.

In line with previously published works, sirolimus introduction was associated with: i) a trend for a better eGFR, ii) an increase of cholesterolemia, and a drop in kalemia and phosphoremia (**Table 3**). No case of NODAT was observed during the follow-up period.

**Table 3:** Biological changes associated with sirolimus introduction.

	Day 0	Day 90	p
Creatininemia (µmol/L)	131±31	116±24	0.059
eGFR* (mL/min/1.73 m^2^)	54±17	61±16	0.125
Kaliemia (mmol/L)	4.2±0.4	4.0±0.4	0.119
Phosphoremia (mmol/L)	1.0±0.2	0.8±0.2	0.023
Total cholesterol (mmol/L)	4.8±1.1	5.6±1.2	0.013
LDL-cholesterol (mmol/L)	2.7±1.0	3.3±1.0	0.050
Triglycerides (mmol/L)	1.9±1.4	2.1±1.1	0.638

Results are expressed as mean ± SD.*Estimated glomerular filtration rate (MDRD formula).

The intensity of clinical inflammatory manifestations previously related to sirolimus in the literature [i.e. stomatitis [15], inflammatory skin disorders (including rash and acnea; [15]), arthritis [16], and pneumonitis [18]] was prospectively monitored.

Sirolimus had to be stopped before the first follow-up visit (after 7 days) in one patient (#25, **Figure 1B**), who developed an acute inflammatory syndrome that combined: fatigue, stomatitis, and dyspnea. These clinical manifestations resolved spontaneously following sirolimus withdrawal suggesting that the drug was responsible for the symptoms.

**Figure 2:**
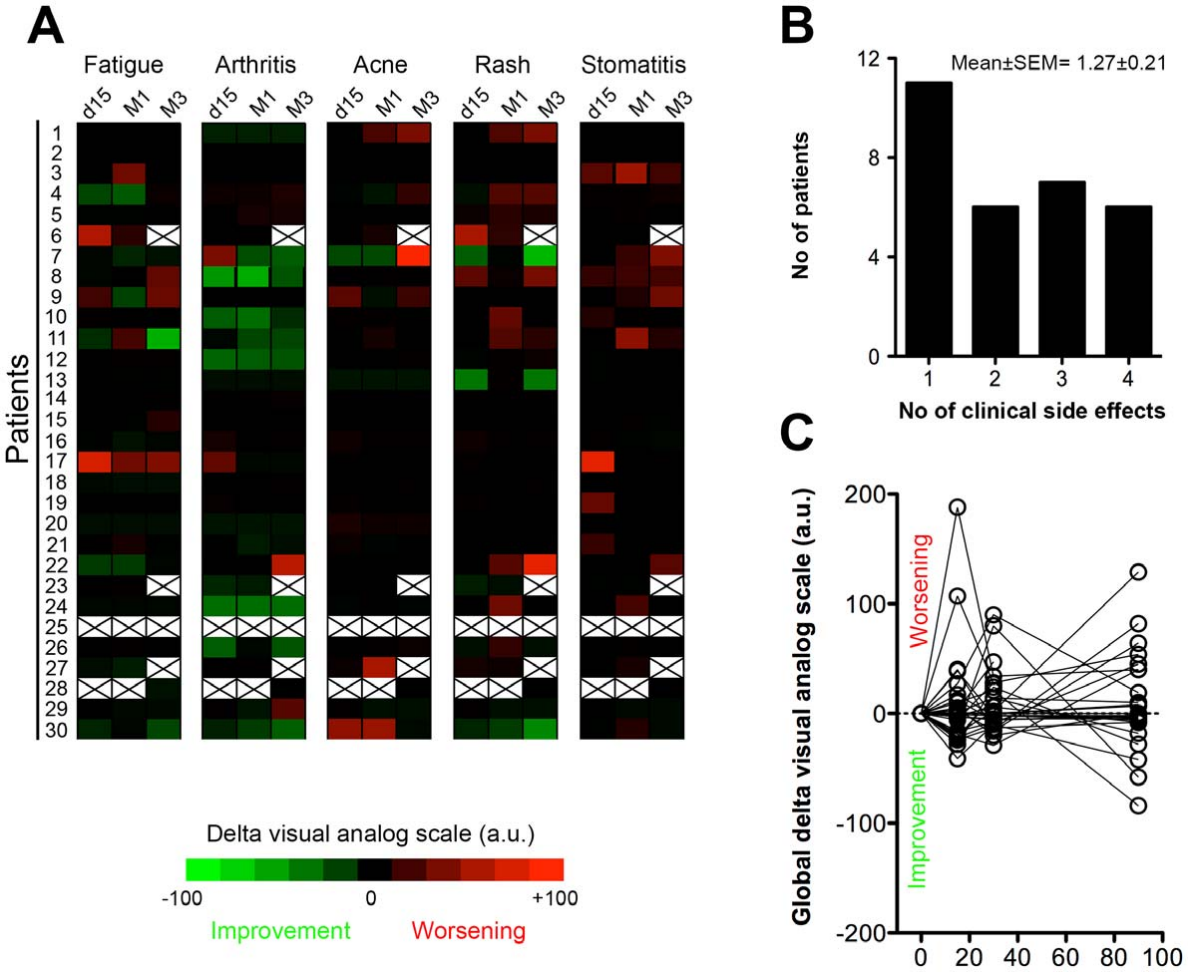
Profile of sirolimus-induced clinical inflammatory syndrome. A) Heat map representing delta visual analogue scale values of every symptom at each time point for the population of the study. Black crosses indicate excluded patients (#6, 23, 25, 27) or missing data (patient #28). B) Histogram showing the distribution of the population according to the number (No) of inflammatory symptoms experienced after the switch over the 3 months follow-up period. C) Evolution of the individual global delta visual analogue scale values over the 3 months follow-up period.

Nineteen of the twenty-nine (66%) remaining patients reported at least one significant clinical inflammatory side effect in the 3 months following sirolimus introduction, and 13/29 (45%) at least 2 **(** **Figure 2A & 2B** **)**.

Cutaneous symptoms were the most frequent, affecting half of the patients (14/29, 48%). Stomatitis was almost as common (12/29, 41%), while fatigue (7/29, 24%) and arthralgia (4/29, 14%) appeared less frequent (**Figure 2A**). The intensity of the symptoms was generally mild (**Figure** **2A, 2C**) but some patients presented a more severe clinical picture, occasionally (Pt #25 & #27; 2/30; 7%) requiring drug withdrawal (**Figure 1B** **)**.

Clinically, sirolimus-induced inflammatory syndrome was mostly characterized by its extreme lability: most of the symptoms being mild, transient with a high degree of fluctuation (**Figure 2A & 2C**). The absence of clear pattern of evolution and the asynchronicity of the symptoms reinforced the stochastic nature of sirolimus-induced inflammatory syndrome: each patient exhibiting a unique clinical presentation.

### Sirolimus Triggers a Rise of Inflammation-associated Acute Phase Proteins

In contrast with the diversity of its clinical presentations, the biochemical profile of sirolimus-induced inflammatory syndrome was much more homogenous.

**Figure 3:**
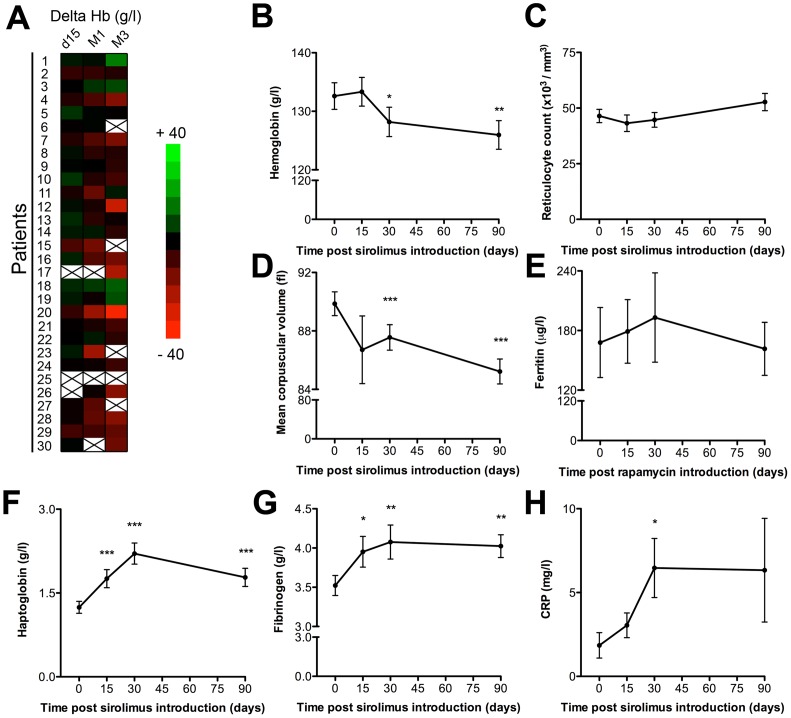
Profile of sirolimus-induced biological inflammatory syndrome. A) Heat map representing delta hemoglobin value at each time point for the population of the study. Black crosses indicate excluded patients (#6, 23, 25, 27) or excluded sera (patient #15, 17, 26, 30). The evolution of the levels of hemoglobin (B), reticulocyte count (C), mean corpuscular volume (D), ferritin (E), haptoglobin (F), fibrinogen (G), and CRP (H) are plotted (mean ± SEM). *p<0.05, **p<0.01, and ***p<0.001, as compared with baseline (d0) value.

In line with previous studies [19], [23]–[26], the vast majority of patients (23/29, 79%) underwent a decrease of hemoglobinemia of at least 5 g/L after sirolimus introduction (**Figure 3A**). The difference of hemoglobin level, already significant one month after the switch (−5.19±1.38 g/L), tended to increase over the follow up period (−7.96±2.38 g/L at 3 months; **Figure 3B**). The absence of bone marrow compensation, as indicated by the stability of reticulocyte count (**Figure 3C**), as well as the drop of the mean corpuscular volume (**Figure 3D**) despite adequate iron store (**Figure 3E**), were both compatible with the diagnosis of anemia of chronic inflammation, in which increased production of hepcidin, a recently identified acute-phase protein [27], prevents ferroportin from releasing iron stores [28]. Quantification of circulating mature hepcidin is currently hampered by technical difficulties [26], [29] and was therefore not performed in this study. However, in agreement with our hypothesis, we observed a concomitant rise of the serum levels of 3 others prototypical acute phase proteins: haptoglobin (**Figure 3F**), fibrinogen (**Figure 3G**) and CRP (**Figure 3H**) following sirolimus introduction. The changes of serum levels of the 3 proteins were remarkably synchronous, reaching a peak one month after the switch.

The imputability of these biochemical inflammatory manifestations to sirolimus was further suggested by the demonstration that the levels of CRP (**Figure S1A**) and fibrinogen (**Figure S1B**) remained stable for 6 months in a control group of 50 stable renal transplant recipients on a continuous calcineurin inhibitor-based regimen.

The production of acute phase proteins by the liver is tightly regulated by inflammatory mediators, in particular cytokines. We therefore went on investigating the impact of sirolimus introduction on cytokine microenvironment.

### Sirolimus Destabilizes Cytokine Inflammatory Balance

In each patient, the profile of cytokine microenvironment was analyzed before and sequentially after sirolimus introduction.

**Figure 4:**
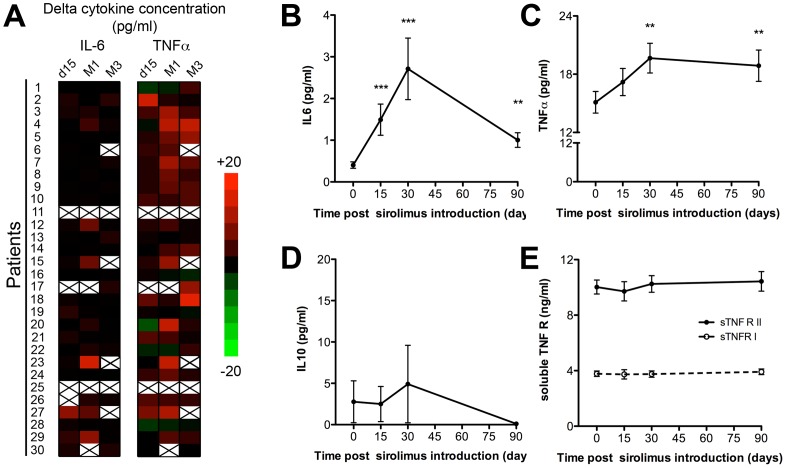
Sirolimus destabilises cytokine inflammatory balance. A) Heat map representing delta cytokine concentration values at each time point for the population of the study. The evolution of the serum levels of IL-6 (B), TNFα (C), IL-10 (D), and soluble TNF receptors (sTNFR) I and II (E) are plotted (mean ± SEM). **: p<0.01, and ***: p<0.001, as compared with baseline (d0) value.

A significant rise of the plasma levels of inflammatory cytokines was observed in the plasma as early as 15 days after the switch (**Figure 4A**). Changes of IL-6 and TNFα levels appeared coordinated, reaching a peak 1 month after sirolimus introduction (**Figure 4B and 4C**).

The physiological down-regulation of inflammatory response involves feed back loops that depend on anti-inflammatory cytokines [30], and receptor antagonists [31]. Interestingly, the increase of the level of inflammatory cytokines induced by sirolimus did not trigger a detectable compensatory rise of anti-inflammatory IL-10 (**Figure 4D**) and soluble TNF Receptors I and II (**Figure 4E**).

**Figure 5:**
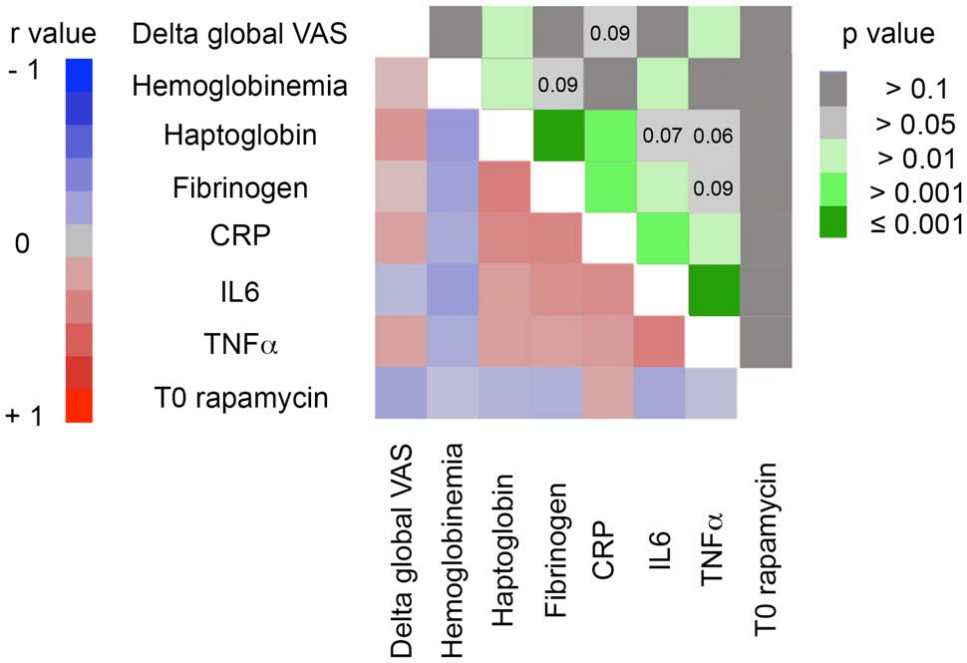
Evolution of inflammatory cytokine levels correlates with clinical and biological manifestations of sirolimus-induced inflammatory syndrome. Pairwise correlation analysis was performed to determine how the changes of inflammatory cytokine levels are related to each other and with the changes of biological and clinical manifestations of sirolimus-induced inflammatory syndrome. The lower left half of the matrix is the color map of correlations. Bright colors indicate the pairs of variables closely correlated in the linear regression model (red for positive and blue for negative correlation). Faded colours encode for decreasing r values. The upper right half of the matrix is the colour map of p values. Decreasing p values are encoded from dark gray to dark green.

In an attempt to evaluate whether the destabilization of inflammatory cytokine microenvironment could account for the biochemical and clinical manifestations observed after sirolimus introduction, we tested the relationship between the variables in a multiple linear regression model (**Figure 5**). Changes of IL-6 plasma level showed significant positive correlation with the ones of fibrinogen (r = 0.31; p = 0.01) and CRP (r = 0.33; p = 0.005), and negative correlation with hemoglobin variations (r = −0.24; p 0.04). Changes of TNFα were highly correlated with the ones of IL-6 (r = 0.42; p = 0.0002) moreover they also positively correlated (r = 0.24; p = 0.04) with the evolution of clinical inflammatory manifestations (**Figure 5**).

We did not find any correlation between sirolimus trough levels and the severity of inflammatory manifestations (**Figure 5**). This finding suggests that the destabilization of inflammatory balance is not a dose-dependent effect of the drug. However, given the small size of the study, one can not rule out the possibility that the lack of statistical power is responsible for this observation.

## Discussion

In the present study, we report the frequent occurrence of paradoxical inflammatory manifestations within the 3 months following sirolimus introduction in kidney transplant recipients.

Symptoms were usually mild, transient, and asynchronous. The stochastic nature of the clinical presentation is likely due to patients’ unique individual backgrounds and sensitivities and offers a probable explanation to why this syndrome has rarely been recognized as a single entity before. Indeed, while sirolimus has been approved by the FDA more than a decade ago, most of the publications related to its safety profile consider each of the clinical inflammatory manifestations as separate side effects rather than resulting from a common pathophysiology.

Biochemical profile of sirolimus-induced inflammatory syndrome was more uniform, mainly characterized by a drop of hemoglobinemia and a moderate rise of acute phase proteins that reached a peak in the serum one month after the switch.

These clinical and biochemical manifestations seem to be the consequences of the destabilization of the inflammatory cytokine balance by sirolimus, the introduction of which correlated with an increase of the levels of inflammatory cytokines IL-6 and TNFα without apparent compensation of the negative feedback loops dependent on IL-10 and soluble TNF receptors.

This pathophysiologic hypothesis is in line with the conclusions of several *in vitro* studies, which report that sirolimus enhances the production of TNFα by macrophages in response to bacterial products by stalling IL-10-dependent inflammatory autoregulation [32], [33].

The molecular mechanism by which sirolimus can differentially modulate pro- and anti-inflammatory cytokine production has been recently elucidated. Activation of mTOR pathway in mononuclear phagocytic cells enhances STAT3 activity and in turn IL-10 production but reduces the activation of the transcription factor NF-κB, the master regulator of proinflammatory responses [34]. Inhibition of mTOR with sirolimus has reciprocal effects [34]. These experimental data have been recently validated in the clinical setting by the observation that the blood transcriptional profile of patients on sirolimus is characterized by an over-representation of genes of innate immune cell lineage and NF-κB pathway [35].

In conclusion, this study shows that sirolimus triggers a disequilibrium of the inflammatory cytokine balance in transplanted patients that leads to a paradoxical inflammatory response with mild stochastic clinical symptoms in the weeks following drug introduction.

This pathophysiologic mechanism unifies the various individual inflammatory side effects recurrently reported with sirolimus and suggests that it would make more sense to consider them as a single syndromic entity.

## Supporting Information

Figure S1 **Changes in serum levels of inflammatory acute phase proteins over 6 months in renal transplant recipients on a continuous calcineurin inhibitor-based immunosuppressive regimen.** The serum levels of CRP (A) and fibrinogen (B), were measured 6 months and 12 months post transplantation in 50 stable renal transplanted patients on continuous calcineurin inhibitor-based immunosuppressive regimen (tacrolimus, n = 39, or cyclosporine A, n = 11). Each patient is an open circle; means are indicated by a black dash. ns: 6 months vs 12 months paired t test, p>0.05.
(TIF)
