# Supplementary figures and images for: Profiling Sirolimus-Induced Inflammatory Syndrome: A Prospective Tricentric Observational Study
Source: PLoS One. 2013 Jan 7;8(1):e53078. doi: 10.1371/journal.pone.0053078 (PMC3538748; doi:10.1371/journal.pone.0053078)

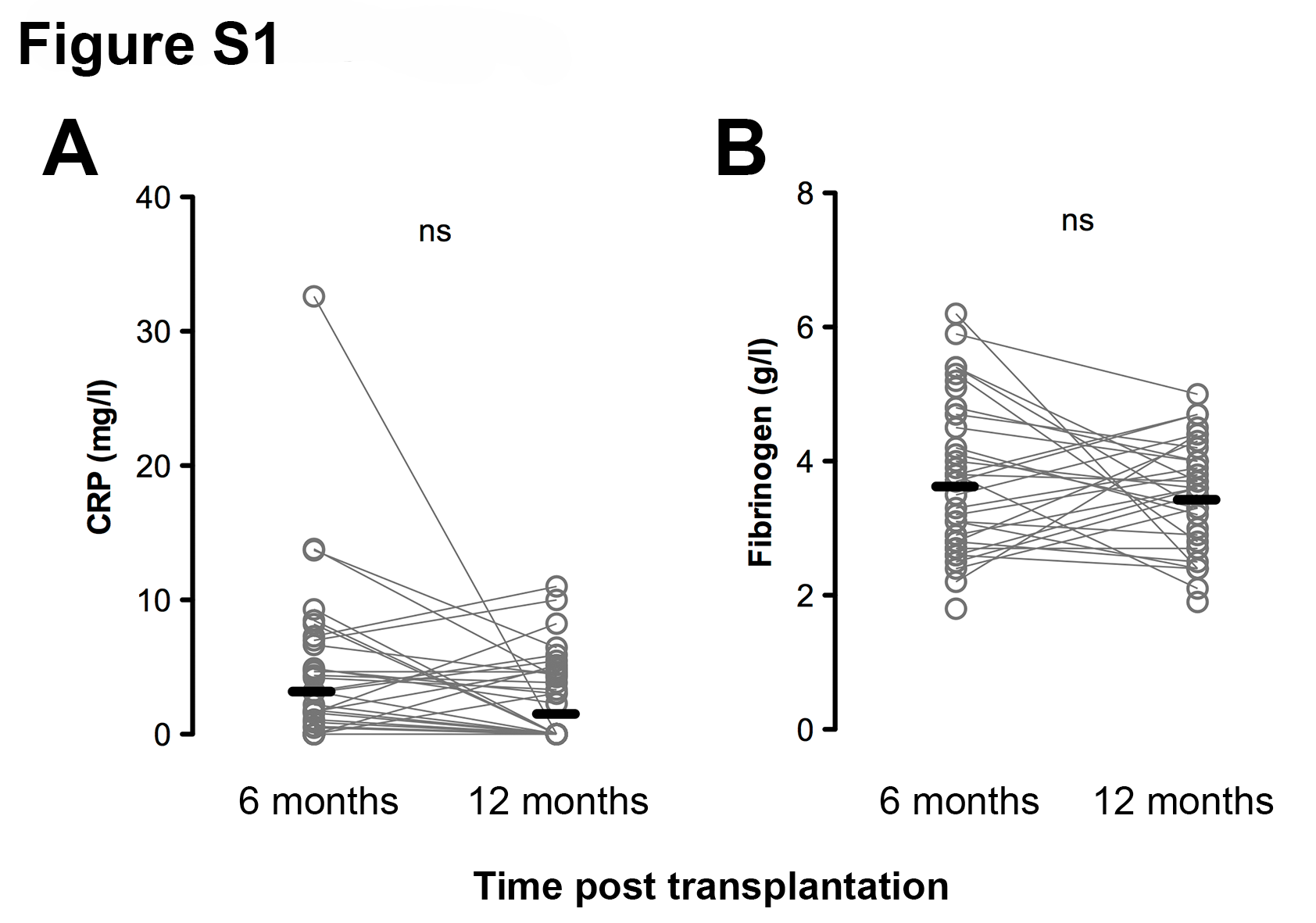

Supplement: Figure S1 — Changes in serum levels of inflammatory acute phase proteins over 6 months in renal transplant recipients on a continuous calcineurin inhibitor-based immunosuppressive regimen. The serum levels of CRP (A) and fibrinogen (B), were measured 6 months and 12 months post transplantation in 50 stable renal transplanted patients on continuous calcineurin inhibitor-based immunosuppressive regimen (tacrolimus, n = 39, or cyclosporine A, n = 11). Each patient is an open circle; means are indicated by a black dash. ns: 6 months vs 12 months paired t test, p>0.05. (TIF) [file pone.0053078.s001.tif]
